# Halitosis and Pain Threshold of Peri-Implant Tissues: An Observational Cohort Study

**DOI:** 10.1590/0103-6440202305527

**Published:** 2023-12-22

**Authors:** João Paulo do Vale Souza, Giovana Dornelas Azevedo Romero, Clóvis Lamartine de Moraes Melo, Lucas Tavares Piacenza, Renata Vasconcelos Monteiro, Emily Vivianne Freitas da Silva, Daniela Micheline dos Santos, Marcelo Coelho Goiato

**Affiliations:** 1 Departamento de Materiais Dentários e Prótese, Universidade Estadual Paulista "Júlio de Mesquita Filho", Araçatuba, Brasil.; 2 Department of Comprehensive Dentistry, University of Louisville, School of Dentistry, Louisville, USA.; 3 Departamento de Prótese da Universidade de São Paulo, São Paulo, Brasil.

**Keywords:** Implant-supported dental prosthesis, halitosis, edentulous mouths

## Abstract

In this study, we aimed to evaluate the halitosis and pain threshold of the peri-implant soft tissues in individuals rehabilitated with implant-supported prostheses. Forty-eight subjects were divided into four groups (n = 12) according to their prosthetic rehabilitation: single-tooth fixed prosthesis, multi-tooth fixed prosthesis, overdentures, and the Brånemark protocol. Halitosis was measured using a halimeter, whereas the pain threshold was measured using Von Frey monofilaments. Measurements were taken before (t0) and 30 days after (t1) placement of healing caps, and at the time of (t2) and 30 days after (t3) prosthetic placement. Halitosis data were analyzed using the chi-square test and Bonferroni correction (p < 0.05). Two-way ANOVA and Tukey’s test (p < 0.05) were used to analyze pain threshold data. We noted an association between halitosis and time for the Brånemark protocol [X2(6) = 18.471; p = 0.005] and overdenture groups [X2(6) = 17.732; p = 0.007], and between halitosis and type of prosthesis only at t0 [X2(6) = 12.894; p = 0.045]. The interaction between time and the type of prosthesis significantly interfered with the mean pain threshold values (p = 0.001). At most time points, the majority of participants in each group had clinically unacceptable halitosis. After 30 days of using the prostheses, the overdenture group had a lower pain threshold compared to the Brånemark protocol group.

## Resumo

Este estudo teve como objetivo avaliar a halitose e o limiar de dor dos tecidos moles peri-implantares em indivíduos reabilitados com próteses implantossuportadas. Um total de 48 indivíduos foram divididos em quatro grupos (n=12), de acordo com as reabilitações: prótese fixa unitária, prótese fixa multidentária, sobredentadura e protocolo de Brånemark. A halitose foi medida com um halímetro, enquanto o limiar de dor foi medido com monofilamentos de von Frey. As medições foram feitas antes (t0) e 30 dias após (t1) a colocação das tampas de cicatrização e no momento (t2) e 30 dias após (t3) a colocação da prótese. Os dados de halitose foram analisados por meio do teste qui-quadrado e correção de Bonferroni (p < 0,05). ANOVA de duas vias e o teste de Tukey (p < 0,05) foram usados para analisar os dados do limiar de dor. Observou-se associação entre halitose e tempo para o protocolo de Brånemark [X^2^(6) = 18,471; p = 0,005] e grupos overdenture [X^2^(6) = 17,732; p = 0,007], e entre halitose e tipo de prótese apenas em t0 [X^2^(6) = 12,894; p = 0,045]. A interação entre o tempo e o tipo de prótese interferiu significativamente nos valores médios do limiar de dor (p = 0,001). Na maioria dos pontos de tempos, a maioria dos participantes de cada grupo apresentava halitose clinicamente inaceitável. Após 30 dias de uso das próteses, o grupo overdenture apresentou menor limiar de dor em comparação ao grupo do protocolo de Brånemark.

## Introduction

In most patients, the origin of bad breath is related to the oral cavity ^1^. Regular oral hygiene is important for patients with dental implants and implant-supported prostheses to prevent the accumulation of biofilms on these structures ^2^ ^,^ ^3^. Patients who find it difficult to clean prosthetic components are more susceptible to developing halitosis ^1^ ^,^ ^3^ ^,^ ^4^. Halitosis refers to an unpleasant odor originating from the oral cavity, which may be associated with the presence of caries, materia alba, tongue coating, poorly adapted prosthesis, dry mouth, or periodontal or systemic diseases ^1^. Bacteria with the potential to generate bad odors include Gram-negative bacteria such as *Treponema denticola, Porphyromonas gingivalis, Porphyromonas endodontalis, Prevotella intermedia, Bacteroides loescheii, Enterobacteriaceae, Tannerella forsythia, Centipeda periodontii, Eikenella corrodens,* and *Fusobacterium nucleatum* ^5^. Metabolism of food debris by these microorganisms produces volatile sulphur compounds (VSC; e.g., hydrogen sulphide, methyl mercaptan, and dimethyl sulphide), which play a key role in the onset of halitosis ^5^ ^,^ ^6^.

The following three methods are considered acceptable to measure and assess halitosis: the organoleptic method, gas chromatography, and sulfide monitoring ^6^. Organoleptic assessment includes the use of the human sense of smell and is a simple ^6^, subjective approach; however, it is difficult to standardize the results due to its subjectivity ^6^. Gas chromatography can separate and quantitatively measure the gases related to bad breath ^6^. Although the results obtained using this method are highly objective and reproducible ^6^, gas chromatography is unsuitable for clinical use as it is expensive and requires experienced operators ^6^. Halimeters are inexpensive and easy-to-use portable VSC meters and have emerged as a solution between the two aforementioned extremes ^6^.

Loss of teeth removes periodontal receptors and damages nerve fibers, thereby severely damaging the feedback pathway to the brain ^7^. Additionally, after tooth loss, the alveolus is filled with bone, and the other receptors present in the gingiva, joint, muscle, mucosa, and bone assume functions of the periodontal ligament receptors ^7^. The human body contains different types of receptors, including chemoreceptors, mechanoreceptors, thermoreceptors, nociceptors, and photoreceptors, each of which responds to a different stimulus ^8^. According to Jacobs et al. (2002), “In the oral cavity, the somatic and gustatory sensory systems predominate ^8^. The former responds to thermal, mechanical, and nociceptive stimuli, while the latter is sensitive to chemical stimuli ^8^.”

Von Frey monofilament (also called Semmes-Weinstein monofilament) can be used to assess peripheral nerve function by detecting pressure or pain threshold across the entire body, including the skin (without hair), lips, tongue, and oral mucosa ^9^ ^,^ ^10^ ^,^ ^11^. Von Frey filaments are calibrated nylon monofilaments that apply different forces, such as to the skin or mucosa (10.11). Von Frey filaments are one of the most widely used, reliable, non-invasive, and inexpensive methods to quantitatively measure oral tactile sensitivity (mucosa and gingiva) through stimulation of mechanoreceptors such as Meissner corpuscles, Merkel cells, Ruffini endings (touch perception), and nociceptors (pain perception) ^8^ ^,^ ^9^ ^,^ ^10^ ^,^ ^11^ ^,^ ^12^ ^,^ ^13^. According to Jacobs et al. (2002), “Most nociceptors are supplied by Ad and C-fibers. Depending on the stimuli, which can induce pain perception one can identify three types of nociceptors: (i) mechanical nociceptor, (ii) thermal nociceptor, and (iii) polymodal nociceptor” ^8^.

According to the literature, there are no articles comparing halitosis or sensitivity of the oral mucosa to pain using the Von Frey method in users of different implant-supported prostheses. Accordingly, we aimed to evaluate halitosis and the pain threshold of the peri-implant tissues in individuals who were rehabilitated with implant-supported prostheses at four-time points (before and 30 days after placement of the healing caps, and at the time of and 30 days after placement of the prosthesis). The null hypothesis tested was that the type of implant-supported prosthesis would not affect the patient’s level of halitosis and pain threshold of the peri-implant tissues.

## Materials and Methods

### Study design and ethical approval

This non-randomized observational cohort study was conducted by the STROBE guidelines for reporting observational studies ^14^. The two operators (one who measured halitosis and the pain threshold) and another who conducted the statistical analysis were blinded to the study.

After approval by the Human Research Ethics Committee of the Araçatuba School of Dentistry of São Paulo State University (FOA/UNESP - Number: 12730519.4.0000.5420), 48 volunteers were selected according to the inclusion and exclusion criteria outlined below.

All volunteers were rehabilitated at the Graduate Studies Clinic of the FOA/UNESP. The same team performed Prosthetic-surgical planning and implant placement.

### Inclusion criteria

Age between 18 and 80 years, partially or totally edentulous for at least 2 years, systemically healthy, with good oral health, and cognitive ability, and with osseointegrated dental implants for at least 6 (maxilla) or 4 months (mandible). Additionally, only participants who required an all-acrylic overdenture (4 maxillary or 2 mandibular implants), Brånemark acrylic protocol (6 maxillary or 5 mandibular implants), a single-ceramic prosthesis (1 implant in the maxilla or mandible), or a 3-element bonded multiple ceramic prosthesis (supported with 2 or 3 implants in the maxilla or mandible) were included. Furthermore, the participants must not have used any type of removable prosthesis during the osseointegration period and have not undergone any gingival conditioning with implant-supported prosthesis. All patients who were rehabilitated with the Brånemark protocol or overdenture must have used a conventional complete denture over the arch that would be rehabilitated for a period of 2 to 5 years.

### Exclusion criteria

We excluded patients with parafunction and those with harmful habits and addictions, such as drug, alcohol, and tobacco use; and patients with self-reported systemic conditions (diabetes, pregnancy) or who were taking medications that alter bone metabolism (corticosteroids, cyclosporine A, radiotherapy), beta-blockers, or medications that may cause dry mouth.

### Groups

All implants (Biofit DSP, Campo Largo, Brazil) used in this study were the 4.1 external hexagon platform-type, and the diameter and height ranged from 3.75 mm to 5 mm and 10 mm to 11.5 mm, respectively. All implants were placed following principles in the literature ^15^ ^,^ ^16^.

The 48 volunteers selected for the study were divided into four groups (n = 12) according to the prosthetic rehabilitation planned for each patient. Participants received only one prosthesis according to their respective group:

- Single-tooth fixed prosthesis group: Patients were rehabilitated with a single implant that subsequently supported a single screw-retained ceramic crown.
- Multi-tooth fixed prosthesis group: Patients were rehabilitated with two implants that later supported a prosthesis with two or three bonded ceramic crowns. Each implant supported a ceramic tooth, and this type of prosthesis was not of the cantilever type. Additionally, some patients were rehabilitated with three implants that later supported a prosthesis with three bonded ceramic crowns.
- Overdenture group: Patients were rehabilitated with four implants (maxilla) or two implants (mandible). Captures of female parts were performed in the laboratory with thermopolymerizable resin. All overdentures were of the clip-bar type with an O-ring at each of the posterior ends of the metal bar.
- Brånemark protocol group: Patients were rehabilitated with six implants (maxilla) or five implants (mandible). The part of this prosthesis that was close to the mucosa was made of polished metal.

The overdentures and Brånemark protocol prostheses were made of thermopolymerizable acrylic resin (Classico, Brazil) and Trilux artificial teeth (VIPI, Pirassununga, São Paulo, Brazil).

All prostheses were fabricated according to literature protocols ^15^ ^,^ ^16^. All patients received instructions on hygiene based on the type of implant-supported prosthesis. Adjustment of the overdenture acrylic base was performed according to Goiato et al. ^17^. Tongue hygiene was recommended for all patients with a toothbrush or tongue scraper. It is noteworthy that the use of Waterpik was recommended for users of the Brånemark protocol.

### Tests at four-time points

After a period of 4 (mandible) or 6 (maxilla) months of osseointegration, halitosis and Von Frey tests were initially performed at t0 (implants still covered with mucosa and closed with cover screws). Following the data collection at t0, healing caps were placed on the implants by surgically exposing the mucosa. Thirty days after the placement of the healing caps (t1), the aforementioned tests were repeated. Following the data collection at t1, the fabrication of dental prostheses began. All prostheses took approximately four weeks to be fabricated, and immediately following this period prostheses were inserted, accompanied by the repetition of the previous tests (t2 - assessment on the day of insertion of prosthesis). Thirty days after the insertion of the prosthesis, the tests were repeated once again (t3).

Therefore, at t0 and t1, the halitosis and Von Frey tests were performed without using any type of provisional removable prostheses. In contrast, during t2 and t3, these evaluations were performed with the newly inserted implant-supported prostheses. It is worth mentioning that the tests were performed with the prostheses placed inside the patient's oral cavity, except in the case of the overdenture group. For this group, adjustments were initially made to the acrylic base and occlusion. Subsequently, the overdenture was removed to conduct the Von Frey test. This was done because the overdenture covered part of the mucosa where the test was to be conducted.

### Halitosis test

Halitosis was measured using a portable halimeter (HC-312F Tanita Portable Fitscan Breath Checker, Tanita; Arlington Heights, Illinois, USA)., which evaluated the quality of the volunteer’s breath through semiconductors that can measure the number of VSCs emitted by bacteria to classify the odor ^1^ ^,^ ^18^. Before examination, the volunteers were instructed not to drink alcoholic beverages, eat, or chew gum ^1^. Volunteers were also asked to avoid the use of perfumes, mouthwash, shaving creams, lipsticks, or any other cosmetic product, and keep their mouths closed for 10 min (manufacturer’s recommendation) before the measurements were taken ^1^. After examining each patient, the air opening was cleaned with a dry cloth and the halimeter was gently shaken four to five times in the air to remove any odors or moisture left in the product ^18^. Measurements were performed three consecutive times for each volunteer. The volunteers were instructed to blow into the device according to the manufacturer’s recommendations. The device was brought close to the patient’s face until the sensor was at a distance of approximately 1-cm from the patient’s open mouth ^1^ ^,^ ^18^. After the volunteer blew into the device, its display showed the level of halitosis (range, 1−5; 0: no odor; 1: little odor; 2: moderate odor; 3: heavy odor; 4: strong odor; or 5: intense odor), according to the number of VSCs present in the volunteer’s breath.

### Von Frey test (pain threshold)

Pain threshold was measured using Von Frey monofilaments (Semmes-Weinstein monofilaments, North Coast Medical, Inc., Morgan Hill, CA, USA), comprising 20 monofilaments of the same length but with different diameters. According to the manufacturer, the filaments are individually calibrated to provide a target strength, from 0.008 to 300 g.

In this test, the operator placed the tip of a filament on the buccal mucosa around the implant and gradually increased the force on the filament until it bent, at which point, the filament was held in position for 1.5 s and then removed. After removing each filament, the volunteers were instructed to answer “yes” if they experienced pain or “no” if they did not. The test was conducted in a quiet environment to help the patient pay attention to the procedure. The filaments were used in ascending order based on their thickness. This test was performed with the patient in the supine position on the dental chair. In case of a negative response, a subsequent filament with a larger diameter was applied until the volunteer provided a positive response. Following a positive response, the evaluator recorded the number on the final filament tested in a spreadsheet for statistical analysis of the data. These precautions were followed according to the manufacturer of the filament.

### Statistical analysis

SPSS software (version 24.0, SPSS Inc., Chicago, IL, USA) was used to conduct the statistical analyses. For the analysis of halitosis data, the levels were grouped into pairs (no and little odor; moderate and heavy odor; and strong and intense odor). Chi-squared test was performed to verify the association between the halitosis level and the time of analysis, and between the halitosis level and the type of prosthesis. To identify potential differences arising from the chi-squared test, Bonferroni correction was used to change the level of significance to avoid errors derived from multiple comparisons. The corrected significance level was p = 0.004 for the conditions in which 12 analyses were performed (Halitosis level: 3 odor levels and 4 times; 3 odor levels and 4 prostheses), and p = 0.006 for the conditions in which eight analyses were performed (Halitosis level: 2 odor levels and 4 prostheses).

The number of implants was correlated with the level of halitosis in general and at each analysis time point using Kendall's correlation. All analyses were performed at a 5% significance level.

The pain threshold data for each type of prosthesis measured at different times were subjected to a two-way ANOVA, followed by Tukey’s test (*p* < 0.05).

## Results

Forty-eight volunteers of different sexes (27 men and 21 women) and ages (mean age: 49.40 years) participated in this study and were rehabilitated using four types of prostheses: Brånemark protocol (eight men and four women; mean age: 65.64 years), overdenture (six men and six women; mean age: 57.38 years), a single-tooth fixed prosthesis (nine men and three women; mean age: 37.88 years), and multi-tooth fixed prosthesis (four men and eight women; mean age: 36.72 years). The number of prostheses per arch in each group was as follows: Brånemark protocol (maxilla: 5/mandible: 7), overdenture (mandible: 12), single-tooth (maxilla: 6/mandible: 6), and multi-tooth (maxilla: 3/mandible: 9). The multi-tooth group included four rehabilitations with three implants (mandible) and eight rehabilitations with two implants (maxilla and mandible). In the study, there were no dropouts and 100% of the implants placed were successful.

An association was observed between the halitosis level and the time of analysis for the Brånemark protocol [X^2^(6) = 18.471; *p* = 0.005] and overdenture groups [X^2^(6) = 17.732; *p* = 0.007] (Table 1). In the Brånemark protocol and overdenture groups, the count that demonstrated statistical significance was strong or intense odor at t3. The strong or intense odor was less frequent at t3 when compared to other halitosis levels (*p* < 0.05; Table 1), although this was not observed for the single-tooth prosthesis [X^2^(6) = 12.308; *p* = 0.055] or multi-tooth prosthesis [X^2^(6) = 9.024; *p* = 0.172] (Table 1).

Table 2 presents the distribution of volunteers based on the different types of prostheses for each time of analysis. An association was observed between the halitosis level and the type of prostheses only at t0 [X^2^(6) = 12.894; *p =* 0.045]. At t0, the count that exhibited statistical significance was moderate or heavy odor for the Brånemark protocol group. For the Brånemark protocol group, moderate or heavy odor was more frequent at t0 compared to other groups (*p* < 0.05). However, the association between the halitosis level and the type of prostheses was not observed at t1 [X^2^(6) = 10.549; *p =* 0.103], t2 [X^2^(6) = 8.981; *p =* 0.175], or t3 [X^2^(3) = 0.354; *p =* 0.950].

**Table 1:** Level of halitosis of the study participants according to the time analyzed for the different types of prostheses

	Brånemark protocol					Overdenture					Single-tooth					Multi-tooth				
	Time (t)				X^2^	Time (t)				X^2^	Time (t)				X^2^	Time (t)				X^2^
Halitosis level	0	1	2	3	0.005	0	1	2	3	0.007	0	1	2	3	0.055	0	1	2	3	0.172
No odor and little odor (0-1)	0	0	0	2		4	0	0	4		0	2	0	3		1	0	1	2	
Moderate and heavy odor (2-3)	11	8	5	10		7	11	7	8		9	9	7	9		7	11	10	10	
A strong and intense odor (4-5)	1	4	7	0		1	1	5	0		3	1	5	0		4	1	1	0	

X^2^: Chi-squared test

When analyzing the correlation between the number of implants and the level of halitosis in general, no correlation was observed (*p* = 0.274). When correlation was performed at each time point, a positive correlation (correlation: 0.297; *p* = 0.024) was observed only at t1, while no correlation was observed at t0 (*p* = 0.432), t2 (*p* = 0.575), or t3 (*p* = 0.455).

Concerning the pain threshold for each type of prosthesis at different analysis times, a statistically significant difference was detected in the “type of prosthesis × time” interaction (two-way ANOVA: *p* = 0.001). The pain threshold values were significantly different between the overdenture and Brånemark protocol groups and the single-tooth prosthesis and Brånemark protocol groups at t1, and between the overdenture and Brånemark protocol groups at t2 and t3 (Table 3). Individual assessments of each prosthesis revealed significantly lower pain threshold values for single-tooth prostheses at t1, t2, and t3, and for overdentures and multi-tooth prostheses at t3.

## Discussion

In this article, we aimed to evaluate halitosis and the pain threshold of the peri-implant tissues in individuals who were rehabilitated with implant-supported prostheses at four-time points (before and 30 days after placement of the healing caps, and at the time of and 30 days after placement of the prosthesis). The null hypothesis was rejected because the type of implant-supported prosthesis significantly affected the patient’s level of halitosis and the pain threshold of peri-implant tissues.

It has been reported that edentulism may be related to people older than 50 years ^19^. By evaluating the demographic data of this study, it is possible to corroborate this statement, as users of the Brånemark protocol or overdenture had a mean age of > 50 years. In contrast, those who used the other types of prostheses had partially dentate arches and were < 50 years old.

Despite the statistical results, halitosis values from 0 to 1 are clinically acceptable, while those from 2 to 5 are considered unacceptable ^18^. Thus, at most time points, the majority of participants in each group had clinically unacceptable halitosis (Tables 1 and 2). This showed that, based on a clinical acceptability analysis, unacceptable halitosis occurred regardless of the prosthesis type or material; this is probably due to failure to clean the tongue ^1^ ^,^ ^2^, as the included patients had good oral condition, with no periodontal problems, caries, or oral lesions.

During all stages of this study, patients were motivated to improve their oral hygiene, even before implant placement. This type of motivation is common in patients who receive dental implants ^20^ to control or reduce the plaque index, and prevent mucositis and peri-implantitis ^3^, and may explain the successful osseointegration of the implant in all cases. Despite this, only at t3 (Table 2) did most participants from all groups have clinically acceptable halitosis; this was probably due to the patients’ greater hygiene motivation due to the new aesthetic aspect of their smile. It has been reported that an aesthetic smile can positively influence the quality of life and the psychological factors of patients ^19^, which may have resulted in greater motivation for oral hygiene.

**Table 2:** Halitosis level of study participants according to the four types of prostheses

Time	Halitosis Level	Types of prostheses				X^2^
		Brånemark protocol	Overdenture	Single-tooth	Multi-tooth	
t0	No odor and little odor (0-1)	0	4	0	1	0.045
	Moderate and heavy odor (2-3)	11	7	9	7	
	Strong and intense odor (4-5)	1	1	3	4	
t1	No odor and little odor (0-1)	0	0	2	0	0.103
	Moderate and heavy odor (2-3)	8	11	9	11	
	Strong and intense odor (4-5)	4	1	1	1	
t2	No odor and little odor (0-1)	0	0	0	1	0.175
	Moderate and heavy odor (2-3)	5	7	7	10	
	Strong and intense odor (4-5)	7	5	5	1	
t3	No odor and little odor (0-1)	9	10	9	9	0.95
	Moderate and heavy odor (2-3)	3	2	3	3	
	Strong and intense odor (4-5)	0	0	0	0	

X^2^: Chi-squared test

In this study, only at t1 was there a positive correlation between the number of implants and the level of halitosis (correlation: 0.297; *p* = 0.024). Despite this, this correlation was considered weak ^21^. Thus, based on the present study, it is not possible to consider a correlation between the number of implants and the level of halitosis.

In this study, the single-tooth (37.88 years) and multi-tooth (36.72 years) groups had similar mean ages. Likewise, the Brånemark protocol (65.64 years) and overdenture (57.38 years) groups also had similar mean ages. It has been reported that there is a reduction in the perception of physical touch on the skin and oral mucosa (higher touch threshold) with age, possibly due to a decrease in the number of Merkel cells and Meissner corpuscles (mechanoreceptors) ^11^ ^,^ ^13^ ^,^ ^22^. However, the situation is the opposite about pain, as there is evidence that the perception of pain may increase with aging (lower pain threshold) ^11^ ^,^ ^23^. Changes in nociceptors related to aging may favor increased pain signaling ^23^. Thus, it was expected that the pain threshold would be lower in the Brånemark protocol and overdenture groups (older age groups) compared to the other groups (younger age groups); however, based on the Von Frey test, when comparing the values at t0, t1, t2, or t3, no evidence of this was observed (Table 3).

**Table 3:** Mean and standard deviation of the pain threshold according to the type of prosthesis and assessment time

Time	Brånemark protocol	Overdenture	Single-tooth	Multi-tooth
T0	4.66 ± 0.80^Aa^	4.26 ± 0.59^Aa^	4.74 ± 1.15^Aa^	4.65 ± 0.99^Aa^
T1	4.81 ± 0.75^Aa^	3.98 ± 0.62^BCbc^	3.89 ± 0.78^Cd^	4.62 ± 1.03^ABa^
T2	4.80 ± 0.83^Aa^	4.09 ± 0.58^Aab^	4.30 ± 0.88^Ab^	4.51 ± 0.91^Aa^
T3	4.73 ± 0.75^Aa^	3.85 ± 0.69^Bc^	4.13 ± 0.90^ABc^	4.34 ± 0.86^ABb^

Different vertical lowercase letters represent a statistically significant difference. Different horizontal capital letters represent a statistically significant difference (*P* < 0.05, Tukey’s test ).

Tooth extraction damages peripheral innervation in the area of surgery ^7^ ^,^ ^13^. Therefore, it is possible to consider that the single-tooth group (37.88 years) had better-preserved innervation around the edentulous area compared to that of the multi-tooth group (36.72 years). Comparing these two groups with similar mean ages at t0, t1, t2, or t3, there was no evidence that a more preserved innervation around the edentulous area influenced the pain threshold, given that there was no significant difference among them at most time points (Table 3).

At t3, when comparing the overdenture group with the Brånemark protocol group (similar mean ages), it was possible to verify that the overdenture group had a lower pain threshold value (Table 3). Furthermore, comparing t3 with t0 or t2 for the overdenture group or Brånemark protocol group, only the overdenture group showed a reduction in pain threshold at t3. Theoretically, this result may have occurred for several reasons. I - Due to the impact of the overdenture base on the mucosa during mastication of patients, who still had limited neuromuscular ability to control their new overdentures and bite force ^19^ ^,^ ^24^. These impacts often disordered and with great bite force, may have generated movements of the base of the overdenture that caused damage and inflammation to the mucosa, leading to a reduction in the pain threshold ^25^. It has been reported that the role of inflammatory mediators is a key mechanism underlying peripheral and central pain sensitization ^25^. II - Due to the chronic pressure of the overdenture on the mucosa-associated with the bite force that caused inflammation, with a consequent reduction in the pain threshold ^11^ ^,^ ^13^. III - Due to continuous mechanical stimulation of the overdenture over the mucosa, which caused long-term sensitization of nociceptors in the alveolar mucosa, leading to a reduction in the mucosal pain threshold ^11^ ^,^ ^13^. IV - Due to the compression of the overdenture on the mucosa, which may have reduced epithelial keratinization, generating a mechanically weak mucosa that offered less protection for the nociceptors (increased pain sensitivity) ^22^. Finally, V - due to compression of the overdenture on the mucosa, the thickness of the mucosa was reduced, contributing to the reduction of the pain threshold ^11^ ^,^ ^13^.

When comparing the pain threshold results of the groups studied at t0 or t2, there were no statistically significant differences, likely because none of the participants were wearing any type of removable prosthesis over the implant site; thus, there were no removable prosthesis stimuli that could make the mucosa sore and reduce the pain threshold ^13^ ^,^ ^22^. When comparing the results at t1 between groups, significant reductions in pain threshold were observed for overdenture and single-tooth groups compared to that in the Brånemark group, or for the single-tooth group compared to the multi-tooth group; this was probably due to poor oral hygiene in the region of the implants, which caused accumulation of food between the gingiva and the healing cap, generating local inflammation and, consequently, a decrease in the pain threshold ^25^. As previously reported, there were no significant differences between values at t2, probably due to improved oral hygiene in the implant areas.

The limitations of this study include the absence of an evaluation phase of the participants in each group before implant placement, as well as a low period of prosthesis use of only 30 days. Another limitation is the lack of similar studies in the literature to compare the results. Therefore, more studies of this nature are needed. Furthermore, future studies may evaluate the dental arches separately.
